# Low Back Pain Exacerbation Is Predictable Through Motif Identification in Center of Pressure Time Series Recorded During Dynamic Sitting

**DOI:** 10.3389/fphys.2021.696077

**Published:** 2021-09-14

**Authors:** Ziheng Wang, Keizo Sato, Saida Salima Nawrin, Namareq Salah Widatalla, Yoshitaka Kimura, Ryoichi Nagatomi

**Affiliations:** ^1^Department of Medicine and Science in Sports and Exercise, Graduate School of Medicine, Tohoku University, Sendai, Japan; ^2^Division of Biomedical Engineering for Health and Welfare, Graduate School of Biomedical Engineering, Tohoku University, Sendai, Japan; ^3^Next Generation Biological Information Technology, Graduate School of Biomedical Engineering, Tohoku University, Sendai, Japan; ^4^Artificial Intelligence (AI) in Obstetrics and Gynecology Medical Care, Graduate School of Medicine, Tohoku University, Sendai, Japan

**Keywords:** motif-aware state assignment, probabilistic neural networks, social spider algorithm, low back pain, sitting behavior

## Abstract

**Background:** Low back pain (LBP) is a common health problem — sitting on a chair for a prolonged time is considered a significant risk factor. Furthermore, the level of LBP may vary at different times of the day. However, the role of the time-sequence property of sitting behavior in relation to LBP has not been considered. During the dynamic sitting, small changes, such as slight or big sways, have been identified. Therefore, it is possible to identify the motif consisting of such changes, which may be associated with the incidence, exacerbation, or improvement of LBP.

**Method:** Office chairs installed with pressure sensors were provided to a total of 22 office workers (age = 43.4 ± 8.3 years) in Japan. Pressure sensors data were collected during working days and hours (from morning to evening). The participants were asked to answer subjective levels of pain including LBP. Center of pressure (COP) was calculated from the load level, the changes in COP were analyzed by applying the Toeplitz inverse covariance-based clustering (TICC) analysis, COP changes were categorized into several states. Based on the states, common motifs were identified as a recurring sitting behavior pattern combination of different states by motif-aware state assignment (MASA). Finally, the identified motif was tested as a feature to infer the changing levels of LBP within a day. Changes in the levels of LBP from morning to evening were categorized as exacerbated, did not change, or improved based on the survey questions. Here, we present a novel approach based on social spider algorithm (SSA) and probabilistic neural network (PNN) for the prediction of LBP. The specificity and sensitivity of the LBP inference were compared among ten different models, including SSA-PNN.

**Result:** There exists a common motif, consisting of stable sitting and slight sway. When LBP level improved toward the evening, the frequency of motif appearance was higher than when LBP was exacerbated (*p* < 0.05) or the level did not change. The performance of the SSA-PNN optimization was better than that of the other algorithms. Accuracy, precision, recall, and F1-score were 59.20, 72.46, 40.94, and 63.24%, respectively.

**Conclusion:** A lower frequency of a common motif of the COP dynamic changes characterized by stable sitting and slight sway was found to be associated with the exacerbation of LBP in the evening. LBP exacerbation is predictable by AI-based analysis of COP changes during the sitting behavior of the office workers.

## 1. Introduction

Low back pain (LBP) is a highly common issue (Boisson et al., 2019) among people of all ages (Kamper et al., 2011; Hoy et al., 2012; Swain et al., 2014), and is generally described as pain, muscle stiffness, or rigidity located below the costal margin and above the lower gluteal folds, with or without leg pain (sciatica) (Koes et al., 2006). LBP is a common ailment that affects a large percentage of the population, with a lifetime incidence of 58–84% (Airaksinen et al., 2006; Hoy et al., 2012; for Health Statistics, 2014). Even among adolescents, 37% of the participants reported experiencing LBP monthly or more frequently (Swain et al., 2014). In the coming decades, the global burden of LBP is anticipated to rise even more (Hartvigsen et al., 2018). LBP affects function, societal participation, and personal financial well-being in various biophysical, psychological, and social ways. LBP causes the most disability in working-age people worldwide, especially in low-and middle-income countries where informal employment is common, and job-change options are limited (Hartvigsen et al., 2018). As one of the most common chronic health problems, LBP causes more people to leave the workforce than heart disease, diabetes, hypertension, neoplasm, respiratory disease, and asthma combined (Schofield et al., 2008). People who suffer from this disorder have less wealth than those who do not (Deborah et al., 2015) — when comorbidities are present, this effect is amplified (Deborah et al., 2015). Older people who retire early because of LBP have approximately 87% less total wealth and income-producing assets than those who remain in full-time employment (Schofield et al., 2011).

LBP could be a result of many factors. Its emergence could be attributed to several psychosocial and physical factors. A systematic review showed that structural changes identified by MRI, such as disc bulge, disc extrusion, and spondylolysis, were strongly associated with LBP (Brinjikji et al., 2015a). However, in most cases, the causes of LBP could not be identified and were described as nonspecific (Balagué et al., 2012; Maher et al., 2017). Many imaging (radiography, CT' scan, and MRI) findings in people with LBP were also present in people who did not have LBP (Brinjikji et al., 2015b). Furthermore, LBP risk factors include obesity, age, smoking, and psychosocial factors (such as depression and stress; d'Hemecourt et al., 2000; Deyo et al., 2015). In addition to the above-mentioned causes, static loading in the office environment may worsen LBP (Chou and Shekelle, 2010), and prolonged static sitting was associated with an increased risk of LBP and an increase in LBP over the last 40 years (Anne and Walker, 2002; Eifell et al., 2006; Nidhi et al., 2015; Vos et al., 2017; Baker et al., 2018).

Sedentary behavior is a separate class of behaviors characterized by little physical activity or activities that require low energy consumption of <1.5 metabolic equivalent units (Pate et al., 2008). A study on adult sedentary behavior found that sedentary time spent increased with age, full-time employment, and higher education (Leitzmann et al., 2017). A study of 27,637 people aged 15–98 years from 32 European countries showed that the average recorded weekday sitting period was 5.2 h/day (SD 184 min/day) (Bennie et al., 2013). Research conducted in Australia and the UK reported that office staffs spent 68–70% of a workday and 60–63% of a non-workday (Thorp et al., 2012; Clemes et al., 2014). Japanese office workers spent 63% of a workday and 60% of a non-workday sedentary (Kurita et al., 2019). A study of 1,329 sitting workers shows that 201 (15.1%) acknowledged experiencing LBP during the recent week of the survey (Inoue et al., 2015).

However, there exists conflicting evidence regarding the relationship between sedentary behavior and LBP. A system review revealed that LBP was not consistent with a sedentary lifestyle. They have identified eight high quality studies including cohort studies and case-control studies. Except for one cohort study, none of the studies have identified a statistically significant association between sedentary work or sitting at work and LBP (Chen et al., 2009). Sitting for a longer period may result in the development of LBP, but the incidence rate of LBP development largely varied among the studies ranging from 19.19 to 43.59% (Harkness et al., 2003; Yip, 2004). It is believed that this variability may be due to the dynamic nature of LBP. Even for chronic LBP, patients do not suffer from pain all day long.

Researchers have attempted to identify if a subject had chronic LBP during sitting behavior using several techniques, including artificial intelligence (AI). Three studies compared electromyography (EMG) recordings from trunk muscles, spinal positions, and trunk range of motion between chronic LBP patients and control participants (Liszka-Hackzell and Martin, 2002; Gal et al., 2014; Mohammed, 2018). Several other studies have examined the classification of people with chronic LBP and focused on EMG and trunk motion data to identify chronic LBP (Oliver and Atsma, 1996; Magnusson et al., 1998; Hung et al., 2014; Caza-Szoka et al., 2015, 2016; Olugbade et al., 2015; Ashouri et al., 2017; Du et al., 2018; Hu et al., 2018). Although many previous studies analyzed static traits during sitting (O'Sullivan et al., 2012; Boerema et al., 2020), it has been pointed out that the dynamic nature of LBP had not been considered. It was possible that chronic LBP patients were not suffering from LBP at the time of measurement. LBP does not persist all day long or every day — even the same individual at different times of the day has different levels of LBP. The majority of LBP episodes are brief and have little or no consequences. Still, recurrent episodes are common, and LBP is increasingly recognized as a long-term condition with a variable process, not a series of unrelated events (Hartvigsen et al., 2018). LBP in daily life may differ from that reported by the laboratory measures, where most of the research used EMG and body movement in laboratory settings. Measurement and evaluation over prolonged time periods is almost impossible. Therefore, the currently available non-invasive, unobtrusive and contactless method, capable of collecting data from the sensors on the chair may be suitable in a “real-life” setting.

Another limitation in the previous studies focusing on sedentary behavior may be in the assessment of sitting behavior in an aggregate basis, as to determine sitting behavior as a whole. Typically, the evaluation was commonly determined by the total sitting time (Boerema et al., 2020). This conventional approach conceptualizes sitting activity as a person's static characteristics in a single day. Conversely, some studies that have tried to obtain a sitting posture using pressure sensors placed under a chair, armrests, and chair backrests (Cheng et al., 2013; Griffiths et al., 2014; Huang et al., 2017), merely describe the sitting posture, without providing insight into the nature of sitting behaviors. Such approaches to characterize sitting behavior overlook the fact that sitting is a highly complex process. As shown in many studies that sitting behavior contains slight sways and big sways (Makhsous et al., 2009; Sondergaard et al., 2010; O'Sullivan et al., 2013; Chun-Ting et al., 2014). Interestingly, two previous studies reported less frequent postural shifts in individuals with LBP than in healthy individuals (Dunk and Callaghan, 2005; Akkarakittichoke and Janwantanakul, 2017), with just counting postural shifts above a determined threshold level of displacement. A better characterization of sitting behavior in daily life when most of the LBP episodes occur considering the time sequence property of sitting behavior may give us a better understanding of the physiological nature of LBP. Thus, we hypothesize that sitting behavior can be characterized by constant sequences of states derived from various postural changes during sitting. The term “state” refers to an interpretable template that is repeated frequently. In this study, a “state” was calculated by subsequent time-series clustering of changes of center of pressure (COP), representing a specific action during sitting. The term “motif” consists of multiple states, defined as patterns that have similar shape, and yet exhibit nontrivial variability (Saria et al., 2011), which may be able to determine the sitting behaviors on a more granular basis: identifying the definition and nature of each motif, consisting of different states.

There also remains an issue in the complexity of data derived from long term human behaviors, namely sitting in this study. To understand these complex data, each measurement must be labeled as one of the different states. These states are not present as independent events, and the sequence in which they occur is essential. While traditional multiple time-series repetition methods produce several segments of time (Clarkson et al., 2000; Leonardi and Bühlmann, 2016; Nystrup et al., 2016; Hallac et al., 2019), motifs are anticipated to produce multiple cycles of data in multiple time steps. Thus, it is crucial to determine a motif indicating the recurrent events or a sequence of state changes. To this end, we begin by using Toeplitz inverse covariance-based clustering (TICC) (Hallac et al., 2017) to split the sitting data into different states. Further, we defined each motif by motif-aware state assignment in noisy time-series data (MASA) (Jain et al., 2018). MASA iterates by re-assigning the original measurement to the updated states using motifs, thereby allowing previously noisy sequences to make a correlation to match a given motif. This makes MASA even more robust as it allows previously non-correlated sequences to correlate.

In the prediction step, we use a probabilistic neural network (PNN) as the classifier (Specht, 1990), which is a special type of radial basis function that is significantly faster than backpropagation networks (Georgiou et al., 2008). PNN is based on the probability density function (PDF) and Bayesian classifier (Specht, 1990). However, there are two limitations in PNN. (1) Relative inaccuracy when training with a small sample size and (2) lack of evaluation of the importance of the input variables. Therefore, to address these limitations, this study adopts an optimized PNN with two parameters: one is the smoothing parameter, representing the spread of the distribution, and the other is the weight of the input variable, which changes the shape of PDF so that the contour line is no longer circular, but elliptical.

Furthermore, it is crucial to find an optimal value for the weight and the smoothing parameter to enhance the performance of the model. Earlier, trial and error methods were commonly used to select the parameters; however, these methods were time-consuming. Recently, reinforcement learning algorithms have become one of the methods for computing parameter selection (Kusy and Zajdel, 2014). Meanwhile, this parameter estimation problem was shown to be solved by nature-based computing algorithms. With the rapid growth in the size and complexity of modern optimization problems, nature-based computation has gained an increasing attention as an effective tool for optimization. When compared to the traditional optimization techniques, these algorithms were shown to perform well, especially when solving non-convex optimization problems (Hajela, 1990; Mallipeddi et al., 2011). As a population-based metaheuristic algorithm, the social spider algorithm (SSA), a state-of-the-art nature-inspired swarm intelligence algorithm based on social spiders, exhibited excellent global optimization performance on benchmark tests (Yu and Li, 2015). Therefore, whenever the algorithm can locate a relatively small region near the global optimum, SSA was found to be capable. Thus, we used SSA as the optimizer of PNN and inferred the change in LBP using SSA-PNN.

This study aimed to determine whether the motif consisting of different states identified in the COP changes during sitting behaviors may affect LBP exacerbation. We optimized PNN and used SSA as the optimizer, so that we could be able to predict LBP exacerbation based on the COP data collected. Previous studies have established that sitting behaviors may exhibit particular states; thus, we hypothesized that (1) there is a common motif consisting of more than two states, and (2) the motif is related to LBP.

## 2. Method

### 2.1. Study Design

This study is an observational study of office workers to identify daily exacerbation of low back pain occasionally experienced during their work through the analysis of features in sitting behavior by means of time-series recording of changes in the center of pressure using load cells installed on office chairs. The subjective level of low back pain was checked four times daily at the office during the workdays by means of presenting an electronic questionnaire using a tablet PC. The study participants were recruited at a company, the office of which was located at down-town Tokyo, in July 2017 after the approval of the study protocol by the Ethical Committee of Tohoku University School of Medicine. The measurement and data collection were performed from October to December 2017 at the office in a restriction-free environment where the study participants worked according to their real assignment in the company.

### 2.2. Participants

In order to ensure the safety of procedures and to avoid bias on results, the following inclusion criteria were used: healthy office workers who are between 20 and 59 years old when they gave their informed consent were eligible. The exclusion criteria were those who had serious psychiatric, neurological, or musculoskeletal diseases (musculoskeletal disorders) and have been prohibited by doctors from exercising.

### 2.3. Smart Chair

Conventional office chairs equipped with load cells and WiFi data transfer units were provided to the study participants who gave full informed consent to the study protocol. The dimension of the conventional office chair purchased was 52 cm wide, 58.5 cm in length, and 88.5 cm in height with a single column. Four load cells were fixed on a metal plate of 260 mm wide and 250 mm in front-back direction and 3.2 mm thick, in a rectangular formation of 215 mm wide and 200 mm front-back direction so that the geometric center matches that of the metal plate. The bottom surface of the seat frame was firmly attached to the load cells. Each load cell had a capacity of 50 kg driven and was powered by 5 volts (D.C.). The calibration of the “smart-chair” was performed in three steps to ensure validated output signals. Signals obtained without any weight on the seat were used to define the zero level. This was followed by placing round metal plates of either 40 or 80 kg at the geometric center of the seat. A linear relationship was confirmed within the range of zero to 80 kg. The load cells were wired to a Rasberry Pi processor and the data was transferred to through a WiFi unit at a transfer rate of 100 Hz, and the data was stored online at Amazon Web Server. The seat height was adjusted for each study participant so that both feet could stably be placed on the floor with the legs upright in a comfortable position without extra stretching.

### 2.4. Assessment of Sitting Behavior

In order to assess sitting behavior, spatio-temporal changes in the distribution of pressure across the participants' sitting interface were monitored by the smart chair. The sitting behaviors of office employees, such as leaning in various directions, leaving the chair, and swaying, can be adequately represented by COP. Therefore, we use COP to identify the sitting behavior, calculate as the Equation (1):


(1) $$\begin{array}{l} \mathrm{COP}\left(x,y\right)=\frac{A1\left(-1,1\right)+A2\left(-1,-1\right)+A3\left(1,1\right)+A4\left(1,-1\right)}{4}, \end{array}$$

here, the A1 (Front-Left), A2 (Back-Left), A3 (Front-right), and A4 (Back-Right) indicates the values from the sensors. Since this study aimed to identify the motif of sitting behavior instead of the details of physical activities, therefore data were downsampled to 1 Hz, i.e., we cut the sampling rate based on the first timestamp to 1 sample per second.

### 2.5. Assessment of Subjective Symptoms

This study focused on changes in the subjective levels of LBP, neck pain, the feeling of fullness after breakfast, and sleepiness. All the subjective levels were determined using a modified Likert scale of 0 to 10 where 0 represented no pain, hungry, or not sleepy, and 10 represented the worst pain, full, or sleepy. Questionnaires were automatically delivered at regular times every day (9:00, 11:30, 14:00, 17:00) on a tablet PC provided in this study for each participant. Whenever the participants arrived at the office after 9:00, they were asked to answer the questionnaire whenever they started working. Whenever they had to stay in the office after 17:00, they were asked to answer the questionnaire before they left the office. The change in the level of LBP was defined by subtracting the end-of-day score from the morning score. A negative value indicated LBP exacerbation, 0 no change, and a positive value indicated improvement of LBP. The levels of other subjective symptoms were also assessed in a similar manner.

### 2.6. Relevant Features

Thus, in this study, sex, sitting time, motif occurrence rate, level of sleepiness, and satiety after breakfast were selected to classify changes in LBP. These features were shown to be sensitive to shifts in LBP. Previous studies have identified that gender (Grimmer and Williams, 2000), the degree of sleepiness (Miranda et al., 2008; Alsaadi et al., 2010; Kelly et al., 2011), and the feeling of fullness after breakfast is (Kristjansdottir and Rhee, 2002) potentially affect the level of LBP of the participants.

### 2.7. Data Analyses

**Figure 1:**
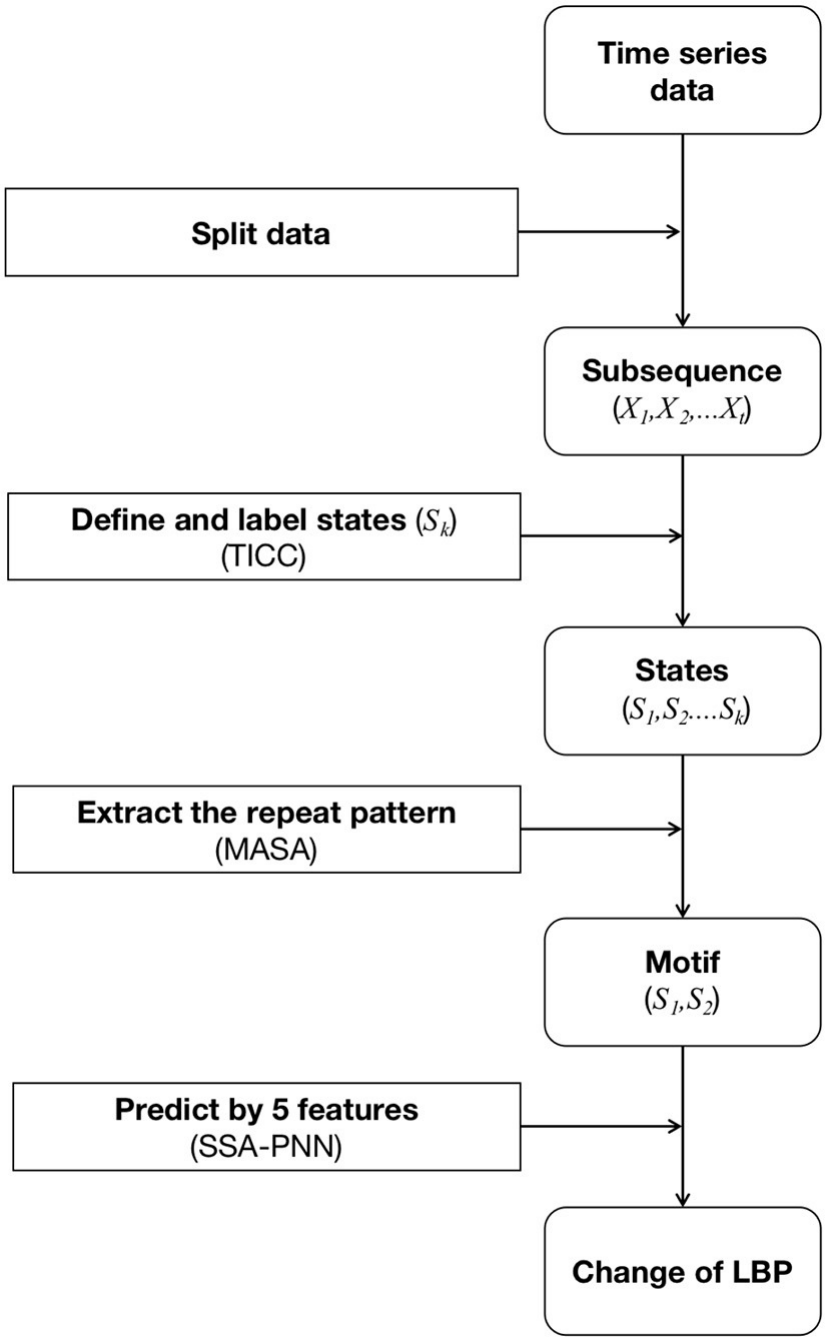
The framework of the feature exaction and the prediction.

In the aforementioned time-series, TICC performs simultaneous segmentation and clustering on the siting behavior data, the motifs were discovered by MASA and prediction by SSA-PNN (Figure 1). The broad collection of time-series data can be represented by a small number of sitting behaviors after these motifs are recognized.

#### 2.7.1. Clustering by TICC

TICC is a model-based subsequence clustering technique for multivariate time-series to discover recurring patterns in temporal data. It assumes that each state (cluster) has a multilayer correlation network, or a Markov random field (MRF) that contains both intra-layer and inter-layer edges, which is specified for each cluster. MRF is a probability distribution model which emphasizes the correlation instead of the distance. Therefore, TICC is not affected by the sitting position. In this study, the states are described as the interrelationships between observations of COP, which can find accurate and interpretable structures of sitting behaviors without the constraint of temporal consistency.

As defined by TICC, the time series of *T* sequential observations,


(2) $$\begin{array}{l} x_{\text{orig}}=\left[\begin{array}{ccccc} | & | & | & |\\ x_{1} & x_{2} & x_{3} & \ldots & x_{T}\\ | & | & | & | \end{array}\right], \end{array}$$

where *x*_*i*_ ∈ *R*^*n*^ is the multivariate *i*-th observation. The objective is to cluster these *T* observations into *K* clusters. TICC focuses on clustering of a short size series *w* ≪ *T* which ends at *t*. The *x*_*t*−*w*+1_, ..., *x*_*t*_ observations are built into an *nw*-dimensional vector *X*_*t*_. Therefore, a new sequence from *X*_1_ to *X*_*t*_ is created, which is a helpful medium for each of the *T* observations to provide proper context. The TICC approach therefore does not cluster the observations directly, but clusters these subsequences with *X*_*t*_, ..., *X*_*t*_. Specifically, TICC constrains the Θ_*i*_'s, the inverse covariances, to be block Toeplitz. Thus, each *nw* × *nw* matrix can be expressed in the following form,


(3) $$\begin{array}{l} \Theta_{i}=\left[\begin{array}{cccccc} A^{\left(0\right)} & {\left(A^{\left(1\right)}\right)}^{T} & {\left(A^{\left(2\right)}\right)}^{T} & \cdots & \cdots & {\left(A^{\left(w-1\right)}\right)}^{T}\\ A^{\left(1\right)} & A^{\left(0\right)} & {\left(A^{\left(1\right)}\right)}^{T} & \ddots & \; & \vdots \\ A^{\left(2\right)} & A^{\left(1\right)} & \ddots & \ddots & \ddots & \vdots \\ \vdots & \ddots & \ddots & \ddots & {\left(A^{\left(1\right)}\right)}^{T} & {\left(A^{\left(2\right)}\right)}^{T}\\ \vdots & \; & \ddots & A^{\left(1\right)} & A^{\left(0\right)} & {\left(A^{\left(1\right)}\right)}^{T}\\ A^{\left(w-1\right)} & \cdots & \cdots & A^{\left(2\right)} & A^{\left(1\right)} & A^{\left(0\right)} \end{array}\right], \end{array}$$

where *A*^(0)^, *A*^(1)^,......, *A*^(*w*−1)^ ∈ *R*^*n*×*n*^. *A*^(0)^ sub-block indicates the intra-time partial interdependencies, so that $A_{ij}^{\left(0\right)}$ defines the interrelationship between concurrent values of sensor *i* and sensor *j* (e.g., the change of COP in two directions). TICC's purpose is to solve the *K* inverse covariances **Θ** = {Θ_1_, ..., Θ_*K*_} and get the corresponding point assignment sets ***P*** = {*P*_1_, ..., *P*_*K*_} (*P*_*i*_ ⊂ {1, 2, ..., *T*}). This leads to an optimization problem in which the following function is to be minimized, as (Equation 4):


(4) $$\begin{array}{l} \underset{\Theta \in T,\text{P}}{arg min}\overset{K}{\underset{i=1}{\sum }}\left[\overset{\text{sparsity}}{\overset{︷}{{\left\|\lambda \circ \Theta_{i}\right\|}_{1}}}+\underset{X_{t}\in P_{i}}{\sum }\left(\overset{\text{log likelihood}}{\overset{︷}{-\ell \ell \left(X_{t},\Theta_{i}\right)}}+\overset{\text{temporal consistency}}{\overset{︷}{\beta \;\left\{X_{t-1}\notin P_{i}\right\}}}\right)\right], \end{array}$$

here, T is the set of symmetric block Toeplitz *nw* × *nw* matrices and || λ ∘ Θ_*i*_ ||_1_ is an ℓ_1_-norm penalty of the Hadamard (element-wise) product to incentivize a sparse inverse covariance (where λ ∈ *R*^*nw*×*nw*^ is a regularization parameter). Additionally, ℓℓ (*X*_*t*_, Θ_*i*_) is the log likelihood that *X*_*t*_ came from cluster *i*, β is a parameter that enforces temporal consistency, and {*X*_*t*−1_ ∉ *P*_*i*_} is an indicator function that checks whether neighboring points are assigned to the same cluster. The TICC problem is solved by alternating minimization using a variation of the EM algorithm.

#### 2.7.2. Discovering Motif by MASA

In noisy time-series results, MASA is used for discovering common motifs and leveraging those motifs to assign states to measurements more robustly. It aims to (1) discover motifs in time-series data that recognize important recurring and length-varying trends and (2) assume that these trends require consecutive measures of time. MASA defines a motif as a sequence of corresponding state assignments and provides a sequence of consecutive measurements, where all neighboring occurrences of the same state are combined into one [MASA defines a time-varying hidden Markov model (HMM) to model the entire sequence of measurements *X*]. Therefore, states are ordered in the motif; however, the number of consecutive occurrences of each state can differ between the motif instances. To this end, each motif is represented by a pair (*m, q*), where *m* is the motif, and *q* is a related list of instances of the motif. In the dataset, a motif instance implies the occurrence of the motif. In our system, we implement the following motif constraints:

(1) The m motif must contain at least three states: |*m*| > 2.
(2) At least L times must appear for a motif *m*: |*q*| > *L*.
(3) Motif instances that do not overlap at most one motif can only belong to each measurement.

As motifs with two or fewer states are not very insightful outside the clustering, MASA encourages the first restriction. The runtime is supported by the second constraint—we can save time by exploring motifs that are more frequent. Because we are only interested in frequent patterns, we do not need a motif for every measurement.

Since the states of sitting may occur in a particular sequence (like a motif), MASA is sufficient to obtain the motif from states of sitting behaviors. MASA seeks to solve for Θ (in this method, the state model is defined using the TICC model), *S* and *M*, by optimizing the following objective (subject to the constraints above) as (Equation 5):


(5) $$\begin{array}{l} \underset{\Theta ,S,M}{\text{max}}\overset{T}{\underset{i=1}{\sum }}\left(\log P_{\Theta }\left(X_{i}|S_{i}\right)-\beta \;\left\{S_{i-1}\neq S_{i}\right\}+\log\gamma \;\left\{S_{i}\notin \text{M}\right\}\right)\\ \;+\text{Ψ}\left(\text{M}\right)-R\left(\Theta \right), \end{array}$$

here,*X*_*i*_ is the measurement at time *i*, which has the assigned state *S*_*i*_, and MASA defines the probability *P*_Θ_(*X*_*i*_ ∣ *S*_*i*_). The β term is a hyper-parameter that encourages neighboring measurements to be assigned to the same state. The γ parameter, 0 ≤ γ ≤ 1, defines the cost of not assigning a measurement to a motif instance. Lower γ values indicate a harsher penalty for a measurement that does not conform to any motif. The term Ψ refers to a scoring metric that measures the strength of our motifs based on how often they appear in the dataset. *R*(Θ) is a regularization penalty on the state model parameters Θ,which formulated the problem of motif discovery as a major optimization problem, which was solved using an expectation-maximization approach.

Considering the interpretability of results and the time of maintaining the state, we set the window size as 5 s (five samples). Refer to the paper about the TICC (Hallac et al., 2017) and MASA (Jain et al., 2018). As an empirical criterion for assessing the optimization model and the relevant clustering outcomes, we used the Bayesian Information Criteria (BIC) which is to characterize the information loss of these models relative to the “real model” as (Equation 6):


(6) $$\begin{array}{l} BIC=-2\ln\;\left(L\right)+k\ln\;\left(n\right), \end{array}$$

here, *L* is the maximum likelihood under this model, *n* is the number of data points, and *k* is the number of parameters in the model. Finally, we used strict rules to obtain the states, the number of clusters was 4, the penalty factor β was selected as 50, and the regularization parameter λ was selected as 0.001.

#### 2.7.3. Prediction by SSA-PNN

We proposed an optimized PNN. First, a set of random real numbers is generated for the weights and smoothing parameters from SSA (Yu and Li, 2015). The weights change the shape of PDF so that the contour line is no longer circular, but elliptical. A larger weight indicates that the variable is more important, and the radius of the ellipse in the direction of the variable is smaller. Meanwhile, the performance of the PNN classifier is highly dependent on the smoothing parameter. Second, data were split into six sets — five sets were used as training sets for training the model, and one set of the test dataset was used to evaluate the accuracy and classification effectiveness of the model. In the training set, we further used four of the five sets for training and one set for validation. This process fits five models on different but partially overlapping training sets and applies a set of parameters generated by SSA to these five models simultaneously. Subsequently, it evaluates them on the non-overlapping validation set and uses the cross-entropy from the validation set as loss functions. Compared to a straightforward training/test split, the main benefit of this approach is the built-in cross-validation to obtain parameters with more generalization power capability, and thus, less bias at smaller sample sizes. However, the disadvantage is that it can significantly increase the training time of the model. Finally, SSA obtains the optimal tuning parameter values that can be applied to a fully independent test set to assess the model in an unbiased manner. In addition, if the smoothing parameter of PNN is lower than 0.1, overfitting becomes likely. Therefore, we set a penalty for the smoothing parameter. When the cross-entropy is minimum, we can use the weight and smoothing parameters to obtain the best model.

Therefore, we use the social spider algorithm as an optimizer (Yu and Li, 2015) to search the smoothing parameters and weights in a modified PNN and tune PNN automatically, demonstrating the applicability of a PNN-based model for decision-making in the classification process. We sought to solve for ω and σ by optimizing the following objective subject, as shown in (Equation 7):


(7) $$\begin{array}{l} \begin{array}{c} \omega_{i}^{*},\sigma^{*}=\arg\min\frac{1}{K}\overset{K}{\underset{k=1}{\sum }}L_{k}, \end{array} \end{array}$$

here, ω_*i*_ represents the *i*-th input variable weight, σ is the standard deviation of Gaussian function that is equivalent to smoothing parameter in PNN, *K* is the number of folds in training set, *L*_*k*_ represents the loss function of *k*-th fold. Cross-entropy is a better measure than MSE for classification, as the decision boundary in a classification task is substantial (in comparison with regression); therefore, we used categorical cross-entropy as the cost function, as (Equation 8):


(8) $$\begin{array}{l} \begin{array}{c} L=\frac{1}{N}\underset{i}{\sum }-\overset{M}{\underset{g=1}{\sum }}y_{ig}\log\left(p_{ig}\left(x_{i}|c_{g}\right)\right), \end{array} \end{array}$$

where *x* represents the testing data vectors; *y*_*ig*_ represents the indicator variable (0 or 1), if the category is the same as the category of sample *i*, it is 1, otherwise it is 0. *M* is the number of clusters. The general classification problem is to determine the category membership of a multivariate sample data (i.e., a p-dimensional random vector x) into one of g possible groups *C*_*g*_, *g* = 1, 2, ........., *g*, based on a set of measurements. Generally, the probabilistic density function is the normal probabilistic density function, as shown in (Equation 9):


(9) $$\begin{array}{l} p_{ig}\left(x_{i}|c_{g}\right)=\frac{1}{{\left(2\pi \right)}^{n/2}\sigma^{n}}\exp\left(-\frac{{\left(x_{j}-x_{ij}^{\left(g\right)}\right)}^{2}}{2\sigma^{2}}\right), \end{array}$$

shows that the only manipulating parameter is smoothing parameter. In this study, for smoothing parameter and multi weights, and as we tested,if σ is smaller than 0.1, the training set was over-fitting, then Equation (9) is developed in the form of the Equation (10):


(10) $$p_{ig}\left(x_{i}|c_{g}\right)=\frac{1}{{\left(2\pi \right)}^{n/2}\sigma^{n}}\cdot \frac{1}{l_{g}}\cdot \sum_{i=1}^{l_{g}}\exp\left(-\sum_{j=1}^{n}\frac{{\left(\omega_{j}x_{j}-\omega_{j}x_{ij}^{\left(g\right)}\right)}^{2}}{2\sigma^{2}}\right),$$

here, *n* is the dimension of the input data, that is, the number of attributes, where *x*_*j*_ represents the value of *j*-th input variable in the testing sample; and $x_{ij}^{\left(g\right)}$ represents the *j*-th input variable of the *i*-th sample of Category *g* in the sample base. Determining the class number of new input data is based on the results of Parzen window. Parzen window is the average probability of input data *x*_*j*_ related to all training samples in each class $x_{ij}^{\left(g\right)}$ for n attributes. *l*_*g*_ is the number of training samples that belongs to class g. Finally, the fourth layer determines class of unknown input data with regard to the highest *p*_*ig*_(*x*_*i*_ ∣ *c*_*g*_).

Furthermore, nine commonly used models (Ridge Regression, Linear Discriminant Analysis, Logistic Regression, Support Vector Machine, K Nearest Neighbors, Extreme Gradient Boosting, Adaptive Boosting, Random Forest, Gradient Boosting) were trained to compare the performance. Because of the small sample size, after tuning the hyperparameter, we set fixed hyperparameters for each model (Supplementary Materials, listing 1) and repeat stratified six-fold cross validation 200 times. The surrogate dataset was subjected to the modeling procedure to confirm whether the performance is significantly better than the chance level.

To identify which feature contributed most to predict the change of LBP level, we calculated SHAP (SHapley Additive exPlanations) values (Lundberg and Lee, 2017). SHAP is a game theoretic approach to explain the output of any machine learning model, SHAP values can quantify the contribution that each feature brings to the prediction made by the model, as (Equation 11):


(11) $$\begin{array}{l} \phi_{j}=\underset{S_{F}\subseteq F\backslash \left\{j\right\}}{\sum }\frac{|S_{F}|!\left(|F|-|S_{F}|-1\right)!}{|F|!}\left[f_{S_{F}\cup \left\{j\right\}}\left(x_{S_{F}\cup \left\{j\right\}}\right)-f_{S_{F}}\left(x_{S_{F}}\right)\right], \end{array}$$

where *x* is the values of the input features, *j* is a certain feature (out of total features *F*), *S*_*F*_ indicates all possible subsets without feature *j*, |*S*_*F*_| is the dimension of *S*_*F*_. To compute this effect, a model *f*_*S*_*F*_∪{*j*}_ was trained with feature *j* present, and another model *f*_*S*_*F*__ was trained with feature *j* withheld. In this study, the SHAP “TreeExplainer” algorithm was used to determine the most important feature in predicting the change of LBP level.

### 2.8. Statistical Analyses

The occurrence rate of the common motif in three categories of LBP change was checked for normal distribution and homogeneity of variance. A non-parametric method for comparing two or more independent variables (Kruskal–Wallis test) was applied. When significant differences were detected, the *post-hoc* comparisons (Dunn's test) were performed. The level of significance was determined as *p*-value < 0.05.

In this study, all of the processing, feature engineering, analysis, and visualization were implemented in Python 3.7.1.

## 3. Results

The total number of study participants was 22, and the participants' demographic profile is presented in Table 1. Total number of days recorded was 90 days. Each participant provided records for 4.09 days in average. We classified participants in four categories according to the changes in the levels of LBP in the recorded days. Three subject (13.64%) experienced exacerbation of LBP in all the recorded days, eight participants experienced no change in the level of LBP, six participants experienced both exacerbation of LBP, and no change, five participants experienced exacerbation, no change and improvement of LBP in the recorded days. as shown in Table 2. Furthermore, Table 3 shows the changes in LBP for all samples, revealing that when LBP exacerbated, sitting time is longer than other groups. There is a clear trend of increasing the common motif from LBP. For both level of sleepiness and the feeling of fullness after breakfast, the no-change group exhibited the highest score.

**Table 1:** Demographic characteristics.

Subject (*n*)	22
Total records, days	90
Records/participants, days	4.09 ± 3.57
Gender (male), %	50.00
Age, year	43.41 ± 8.36
Weight, kg	65.06 ± 9.97
Sitting, hours	5.58 ± 1.93
Motif^a^, frequency/30 min	2.00 ± 1.71
Sleepiness (score from 0 to 10)	6.56 ± 1.96
The feeling of fullness after breakfast (score from 0 to 10)	4.27 ± 3.63
E^b^, *n* of participants, %	13.64
NC^c^, *n* of participants, %	36.63
IM^d^, *n* of participants, %	0
E and IM ^e^, *n* of participants, %	0
E and NC^f^, *n* of participants, %	27.27
E and NC and IM^g^, *n* of participants, %	22.73

a *The common motif identified in this study*.

b *In recorded days, how many participants LBP exacerbated*.

c *In recorded days, how many participants LBP did not change*.

d *In recorded days, how many participants LBP improved*.

e *In recorded days, how many participants LBP exacerbated and improved*.

f *In recorded days, how many participants LBP exacerbated and did not change*.

g *In recorded days, how many participants LBP exacerbated, did not change and improved*.

*E, Exacerbated; NC, did not change; IM, improved*.

**Table 2:** Characteristics by the change of LBP in each subject.

	**E**	**NC**	**E and NC**	**E, NC, and IM**
	**(*n* = 3)**	**(*n* = 8)**	**(*n* = 6)**	**(*n* = 5)**
Gender (male), %	33.33	25.00	83.33	60.00
Sitting, hour	5.72 ± 1.55	4.94 ± 1.78	6.60 ± 1.71	5.01 ± 1.82
Motif, times/30 min	1.56 ± 0.30	2.08 ± 1.72	1.57 ± 1.30	2.44 ± 2.05
Sleepiness (score from 0 to 10)	6.67 ± 2.36	7.77 ± 2.04	5.75 ± 1.60	6.34 ± 1.62
The feeling of fullness after breakfast (score from 0 to 10)	8.67 ± 0.47	6.73 ± 3.08	2.28 ± 3.32	3.79 ± 2.86

*E, Exacerbated; NC, did not change; IM, improved*.

**Table 3:** Characteristics by the change of LBP in each day from all participants.

	**E^a^**	**NC^b^**	**E and NC^c^**
	**(*n* of events = 40)**	**(*n* of events = 43)**	**(*n* of events = 7)**
Gender (male), %	60.00	27.91	71.43
Sitting, hour	6.19 ± 1.79	5.09 ± 1.79	5.11 ± 2.43
Motif, frequency/30 min	1.61 ± 1.25	2.07 ± 1.92	3.71 ± 1.59
Sleepiness (score from 0 to 10)	6.20 ± 1.63	6.93 ± 2.21	6.29 ± 1.48
The feeling of fullness after breakfast (score from 0 to 10)	3.45 ± 3.61	5.37 ± 3.50	2.14 ± 1.96

a *In all of the 22 participants, there are total 40 days that their LBP exacerbated*.

b *In all of the 22 participants, there are total 43 days that their LBP had no change*.

c *In all of the 22 participants, there are total 7 days that their LBP improved*.

*E, Exacerbated; NC, did not change; IM, improved*.

The results obtained from the preliminary analysis of BIC are shown in Table 4, where the penalty factor β was 50, and the regularization parameter λ was 0.001. To reiterate, previous research found that there are more than three states from sitting behavior; thus, we set K from 4 to 10. We can infer that 4, with the smallest BIC value, indicates the best number of states. Therefore, in this study, the states of sitting behavior of the office workers were determined to be 4. Otherwise, it may undermine sensitivity and physiological interpretability.

**Table 4:** The BIC values corresponding to each *K*-values (window size = 5, β = 50, λ = 0.001).

**K**	**4**	**5**	**6**	**7**	**8**	**9**	**10**
BIC for states (×10^5^)	12.28	12.67	13.60	13.19	13.25	12.79	12.72
BIC for motifs (×10^5^)	5.45	8.32	8.06	7.88	8.93	8.44	8.71

*BIC, Bayesian information criterion*.

**Figure 2:**
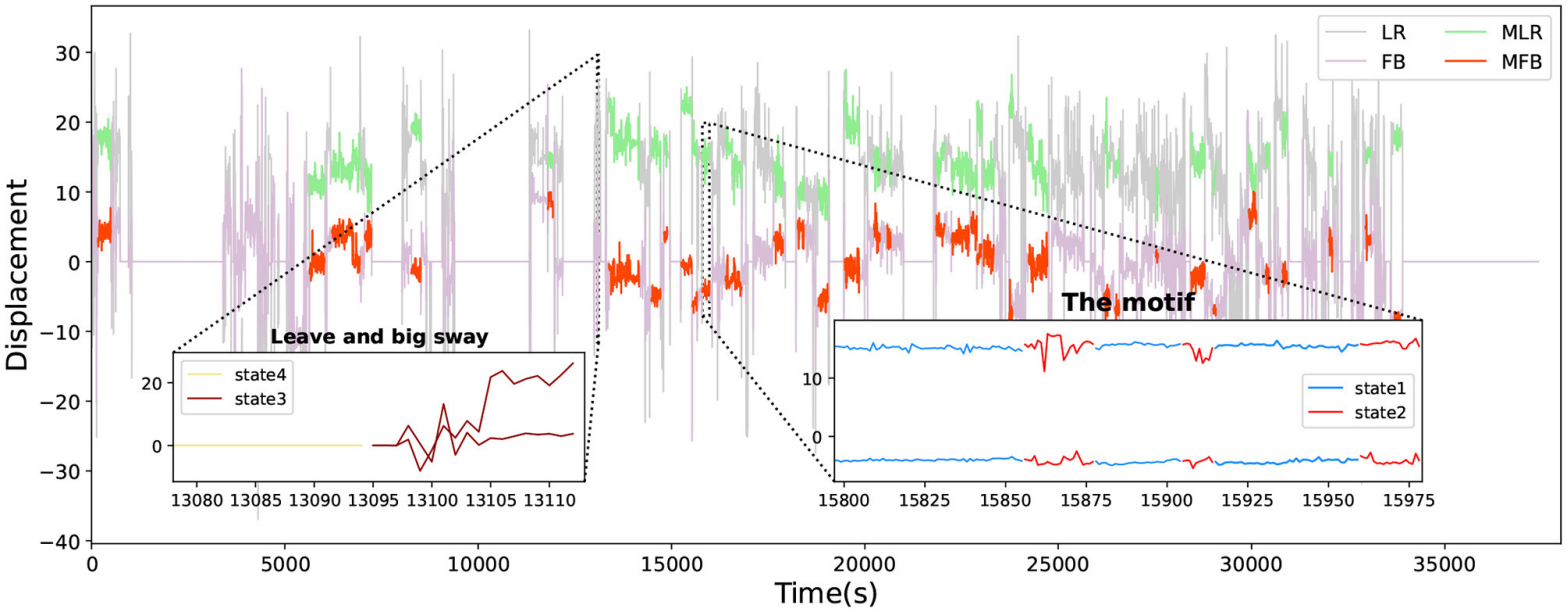
The common motif. We used MASA to extract the most common motif from sitting behavior. LR, the change of COP in left and right; FB, the change of COP in forward and backward; MLR, the change of COP in left and right during the motif; MFB, the change of COP in forward and backward during the motif.

The states of COP are used to reflect the specific pattern from sitting behavior, such as leaving the chair, stable sitting, slight sway, and big sway (Figure 2). Although the states vary slightly among state 1, state 2, and state 3, showing the characteristics of different states of sitting behavior. State 1 indicates stable sitting behavior. State 2 implies slight sway; in general, it is similar to many small actions, such as small stretch or rotation. State 3 indicates big sway. In state 3, the participants moved significantly in both directions. State 4 indicates the participants left the chair. Furthermore, as the figure shows, the common motif consists of state 1 (stable sitting) and state 2 (slight sway), and we found that 91.11% (82/90) of days had this motif. This indicates a series of complex actions that have a specific sequence.

**Figure 3:**
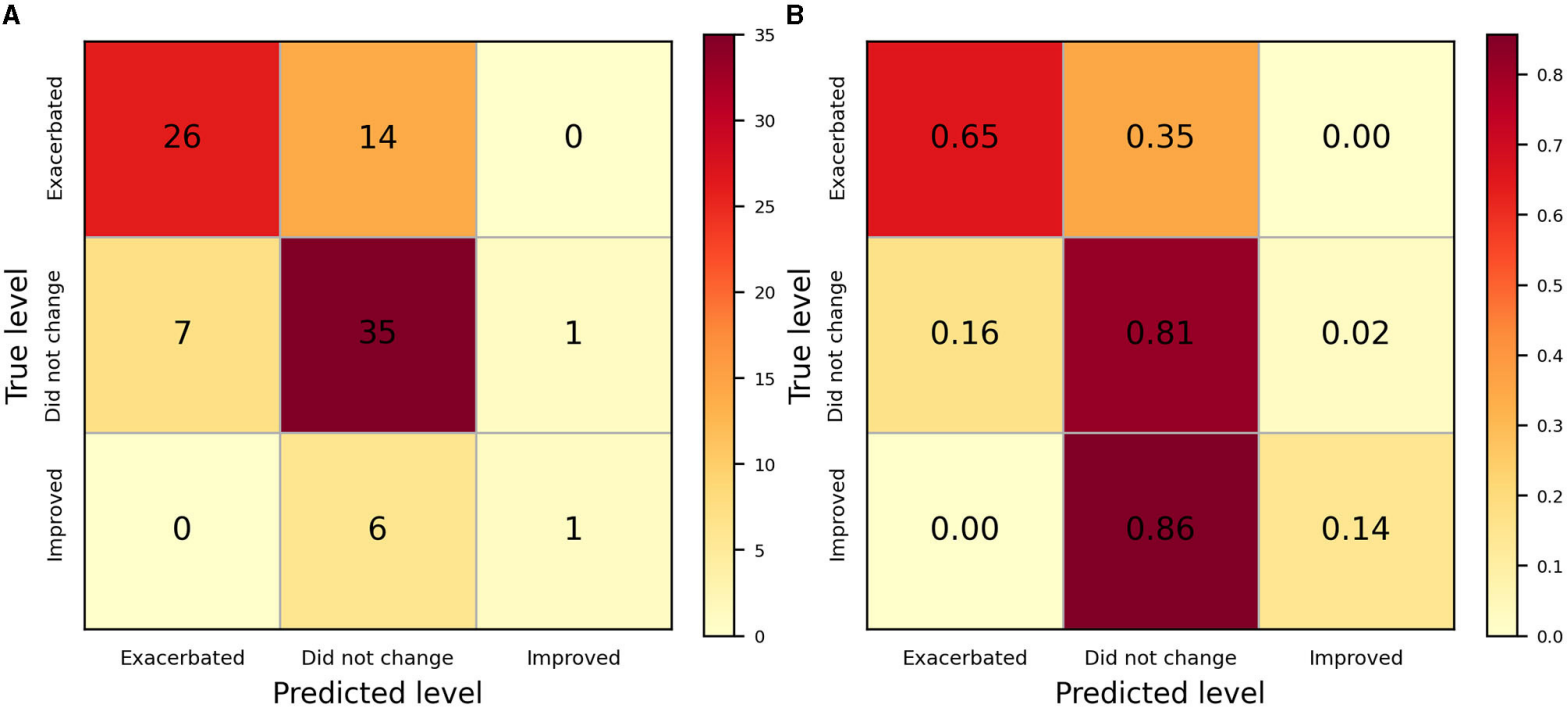
The confusion matrix of SSA-PNN. **(A)** The confusion matrix. **(B)** The normalized confusion matrix. The plots revealed the performance of identifying various levels of LBP, Among them, “did not change” had a better result.

The sitting behavior data were labeled as four states, and a common motif consists of two states. Subsequently, we used the occurrence rate of the motif, sitting time, and other features such as gender, sleepy degree, and how full breakfast was to infer the change of LBP from the morning to night. The output class of the confusion matrix represents the prediction of the PNN-SSA model, enabling it to quickly distinguish confusion between different classes of changes in LBP (Figure 3). In this study, the normalized confusion matrix and confusion matrix were used to achieve a more visual representation. Each matrix column indicates the predicted label at an inference level of LBP, and each row indicates the actual class. The values of the diagonal elements represent the proportions of correct inference levels. Figure 3A shows the number of predictions that are correct, the condition of no change has the highest probability of misclassification. Figure 3B shows the accuracy of SSA-PNN at three levels. SSA-PNN yielded average accuracies of 65, 81, and 14% for worse, no change and better, respectively. The performance of predicting LBP improved was not as good as the other two conditions. This might be attributed to we defined the change of LBP by using the morning score of LBP minus the night score. Most of the differences were very close to 0 which indicates LBP did not change. We hypothesize that the physical conditions of no change and improved are similar, therefore, the accuracy of LBP improved is low.

The results of the performance of the proposed algorithms show a more detailed interpretation, which were evaluated by mean accuracy, weighted precision, weighted recall, and weighted F1 score, as shown in Table 5. Based on data, cross-validation was performed by applying the six-fold cross-validation model for 200 times. In essence, the SSA-PNN and Extreme Gradient Boosting yielded better overall performance on most optimization problems than other algorithms. However, the other methods exhibited very poor classification rates. For this small dataset, SSA-PNN had high levels of classification performance. However, Support Vector Machine had the best recall results compared with the other algorithms (44.32%). Furthermore, we assessed the accuracy (40.74 ± 11.82%) of the surrogate dataset, the performance of SSA-PNN was significantly better than the chance level. In general, SSA-PNN exhibits the best adaptability for small datasets.

**Table 5:** Model performance comparison with fixed hyperparameters.

**Model**	**Accuracy (%)**	**Precision (%)**	**Recall (%)**	**F1 (%)**
SSA-PNN	59.14 ± 10.84	71.82 ± 13.32	41.30 ± 10.89	63.01 ± 10.51
Extreme gradient boosting	58.27 ± 11.67	65.47 ± 13.59	43.73 ± 12.18	60.15 ± 12.14
K neighbors classifier	57.43 ± 11.59	64.44 ± 13.60	43.96 ± 12.81	59.12 ± 12.04
Gradient boosting classifier	57.71 ± 11.82	66.62 ± 14.31	43.44 ± 13.05	60.04 ± 11.98
Random forest classifier	56.99 ± 11.72	66.10 ± 15.00	43.04 ± 12.98	59.32 ± 11.90
Ada boost classifier	56.97 ± 11.69	66.71 ± 14.42	42.25 ± 12.64	59.61 ± 11.78
Ridge classifier	56.69 ± 10.94	64.29 ± 12.48	39.65 ± 9.95	59.22 ± 11.15
SVM—linear kernel	56.51 ± 11.55	62.79 ± 13.44	44.32 ± 13.71	57.88 ± 11.98
Linear discriminant analysis	56.18 ± 11.24	63.24 ± 12.74	41.27 ± 12.72	58.47 ± 11.42
Logistic regression	55.44 ± 11.47	61.71 ± 13.56	43.44 ± 12.91	56.68 ± 11.97

**Figure 4:**
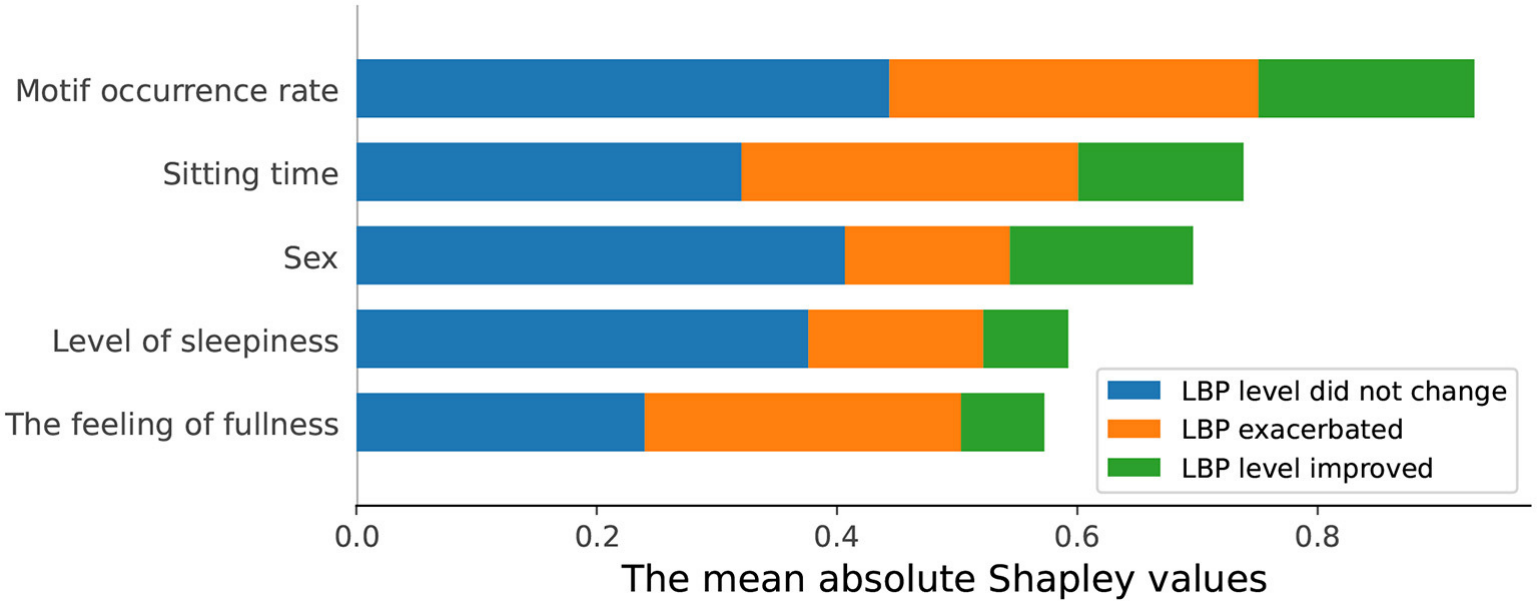
A summary plot of the SHAP values for each feature. The vertical axis indicates the variable names, in the order of importance from top to bottom. The horizontal axis shows the mean absolute SHAP values. The motif occurrence rate contributed the most for predicting the change of LBP level.

The contributions of the features for prediction, as measured by SHAP values, are presented in Figure 4. The SHAP scores in this figure display the contribution to each condition (LBP level did not change, LBP exacerbated, and LBP level improved). The motif occurrence rate had the highest contribution in each condition, demonstrating the best predictive utility.

**Figure 5:**
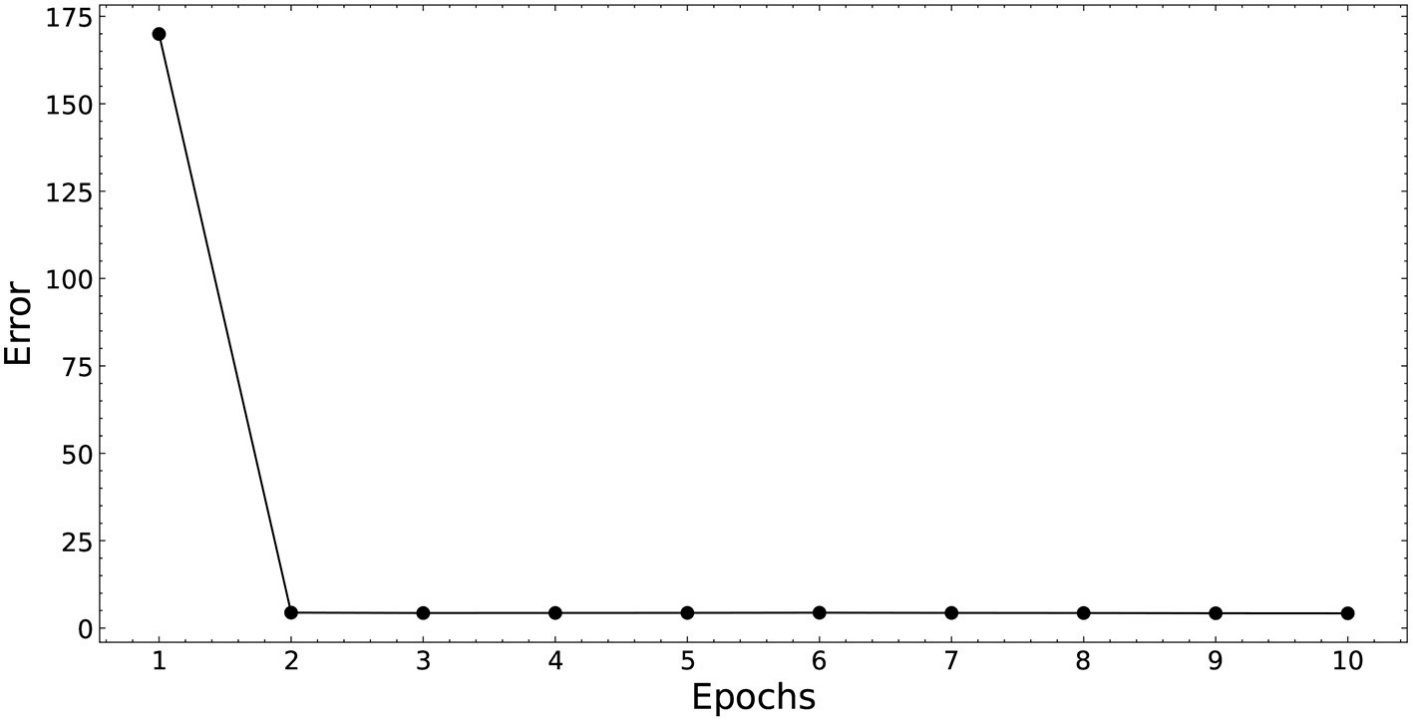
Validation error of SSA-PNN. The plot shows the experimental results of validation with 10 epochs. The best validation performance was attained at epoch 2.

Figure 5 shows the performance of SSA with 10 epochs. It was used as the validation set to obtain σ and ω. In this figure, the smaller the value of the performance, the better the performance of the neural network. There is a considerable gain in performance until the 2nd iteration.

**Figure 6:**
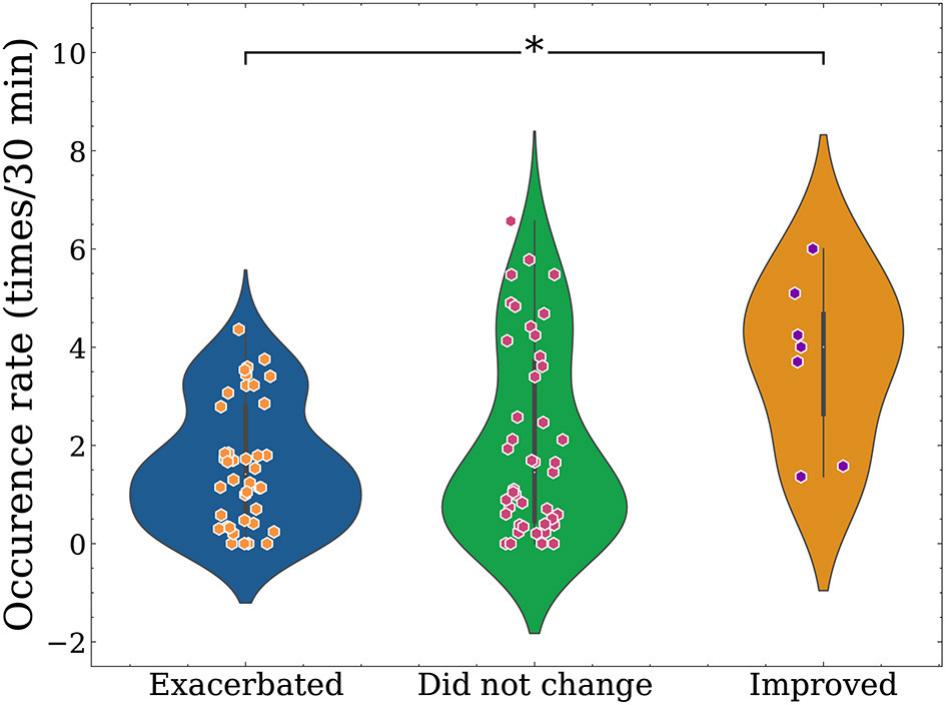
Differences in occurrence rate of motif at three LBP conditions, the figure is the scatter plot with the addition of a rotated kernel density plot on each side. The occurrence rate was higher in the improved group. *p <0.05.

Figure 6 presents the results obtained from the preliminary analysis of sitting behavior. The data did not follow a normal distribution, and the variance was not homogeneous. Significant differences were observed between the three groups (*p* = 0.027; Kruskal-Wallis test), the occurrence rate of the common motif identified in LBP improved was higher compared with worse (*p* = 0.019; Dunn's test) and no change ( *p* = 0.061; Dunn's test).

## 4. Discussion

This study used cross-validation in a new way. In contrast to evaluating the model performance, this study used five-fold to evaluate the generalization of parameters and fixed each set of parameters obtained from SSA to find a set of smoothing and weighting parameters with the best generalization performance on the k sub-dataset. Therefore, the parameter had the best generalization in the five folders. Thus, we used this model to predict the exacerbation of LBP during sitting behavior in real life.

Although no previous studies have examined the motif in sitting behavior, many previous studies have examined the association of sitting behavior with LBP. Recently, ideal workplace sitting posture and sitting behavior have been widely discussed in the literature. The long-standing doctrine of an optimal seating posture that is “as upright as possible” has been highly disputed. The principle of “dynamic sitting” has been slowly substituted, where sitting positions were identified as to continuously change (Dieen et al., 2001; Pynt et al., 2001). However, O'Sullivan in his systematic review concluded that dynamic sitting approaches are not effective as a stand-alone management approach for LBP (O'Sullivan et al., 2012). This conclusion could have been generated by ignoring the potential nature that some individuals have more dynamic sitting behavior (like motif), whereas others have less.

The current study is the first to report the motif of sitting behaviors, which consists of stable sitting and slight sway. The motifs are not the types of sitting posture but a dynamic sitting behavior pattern. This finding might help others to better understand the nature of sitting. The motif is a sitting behavior pattern that lasts <3 min and more than 1 min, it was found to be common for all the subjects in this study. However, it is not clear why this motif occurs. A possible explanation is that when the lumbar spine has too much pressure, human body will produce a non-intentional self-defense mechanism which is a natural physiological behavior, similar to locking the body when one is highly stressed or fatigues, or moving the cervical spine when one gets a neck pain. This will be one of the research directions in future.

Another important finding is that this motif has a positive effect on LBP. These results are consistent with the ideas presented in some review articles of LBP (Dieen et al., 2001; Pynt et al., 2001). Therefore, it suggests that the healthy sitting posture (1) is best thought of as an active, not a static phenomenon, regularly interspersed with moving, (2) is the optimal sitting posture, and (3) helps with lumbar postural health and LBP prevention. In addition, studies have reported less frequent postural shifts in individuals with chronic LBP than in healthy individuals (Dunk and Callaghan, 2005; Akkarakittichoke and Janwantanakul, 2017). Notably, this result may be explained by the shift of stable sitting and slight sway, similar to the movements in the different parts of the trunk muscles, which may alleviate LBP (Dieen et al., 2001). These results corroborate the findings of a previous in which prolonged static contractions of trunk muscles could lead to an increased risk of injury (Nairn et al., 2013).

In contrast, postural modification has been shown to increase the saturation of subcutaneous oxygen, which positively affects tissue viability (Reenalda et al., 2009). Therefore, combined with stable sitting and slight sway, this motif may alleviate LBP. After an in-depth analysis, we found that the motif is always <3 min, which is like a fundamental unit. It can be extended as a longer motif with the same component and sequence. However, we still do not know the mechanism by which sitting behavior exhibits this motif, and we speculate that in unconscious states, the nervous system may be controlling the trunk during sitting behavior for self-protection.

As aforementioned, the motif consists of stable sitting and slight sway that positively affects LBP. These results correlate with a previous study showing that the range of COP displacement in both directions and lumbar curvature were positively correlated with LBP (Sondergaard et al., 2010). First, sitting compresses the intervertebral disc, creating hydrostatic pressure in the nucleus by the annulus and adjoining vertebral bodies (Chan et al., 2011). The amount of hydrostatic pressure within the nucleus is affected by the number of sits (Chan et al., 2011). Therefore, stable sitting and slight sway may be adjusting such pressure. Second, it may be argued that comfortable sitting will preserve lumbar lordosis and transfer the forces acting on the lumbar vertebrae from the intervertebral discs to the lower margins of the articular surfaces of the zygapophysial joints, minimizing the effect of creeping intervertebral discs (Chun-Ting et al., 2014). Third, slight sway shifts a portion of the body weight, thereby reducing the load of back muscles (Makhsous et al., 2009). Following the present results, previous studies have demonstrated that relative to the upper and lower thoracic areas, the non-pain participants displayed a less lateral bent positional shift in the mid-thoracic region. The participants developed transient pain that showed higher muscle activations in the abdominal muscles. In addition, poor to moderate positive associations between rated pain and low back muscle activation were found (Nairn et al., 2013). However, with a small sample size, caution must be applied, as the findings is subject to the selection bias. Thus, it may be inferred that during working hours, stable sitting and slight sway may have a positive effect on LBP.

We also identified two states and many motifs from the sitting behavior. For the other two states, one was absent from the chair; the subjects might have left the chair for lunch or for meetings at a different place. The other state indicated a big sway, which is not the component of the common motif. However, it may indirectly confirm the association between sitting behavior and LBP. Previous research showed that all participants experienced the highest discomfort in the relaxed slouching sitting posture, which is similar to a big sway (Li et al., 2015). As we mentioned, the increase in the degree of variability in the sitting posture is interrelated with the increase in the perceived discomfort (Sondergaard et al., 2010). Notably, this effect may be clarified because those who developed pain had larger L1/L2 intervertebral angles, larger pelvic incidences, and sacral slopes (Misir et al., 2019). In contrast, the flexion-relaxation phenomenon in the relaxed slouching sitting posture caused the body weight to produce mechanical loading on passive tissues (Panjabi, 1992). Furthermore, many motifs consisted of two or more states. Most of these motifs are not as common as the motif we proposed, and we speculate that these motifs highly depend on each individual's characteristic or personality, and there are still several motifs that may reflect LBP. Therefore, it seems that further research can perform clustering based on motifs caused by individual differences to identify a tighter relationship between LBP and such sitting behavior.

Similar to the techniques widely applied to recognize walking, running, and calculating activity consumption, we proposed a novel idea combining machine learning for feature extraction based on dynamic time series of sitting behaviors. Furthermore, we found Recurrent Neural Network (RNN) can learn this motif with accuracy higher than 92% and the motif can be recognized in real-time, this approach solves the problem that TICC and MASA take much time and computer memory to run, this finding should help others to find new ways of applying this tech in practice. However, more feature engineering studies were still needed in this research field. Mapping sitting behavior to a deep feature space may result in some regular features similar to walking or running, we guess some discoveries may be found if chaos theory is integrated into sitting behavior feature extraction.

Despite these promising results, the questions remain. First, our sample of subjects was likely not large enough to represent the population's vast heterogeneity; caution must be applied, as the findings might not be applicable to the entire population. However, application of the same method, it is possible to collect more data and improve the model performance. Second, it is better to use a generative model for data derived from a small sample size. For the bigger data set, discriminate and ensemble models may also have good performance.

## 5. Conclusion

Low back pain exacerbation is predictable through motif identification in center of pressure time series data recorded during dynamic sitting. This study proposed a method of predicting LBP exacerbation of office workers in a “real world” office environment. We split the time-series data of COP changes into four states and used MASA to find out the common motif consisting of stable sitting and slight sway, which may reduce LBP. We used the motif as one of the features to determine the changes in LBP by SSA-PNN, which had better performance compared with the other nine commonly used algorithms. The contribution of this study is to confirm the dynamic nature of sitting behavior, which has significant implications for understanding LBP and sitting behavior. Further studies are required to validate the effect of this motif on LBP; large randomized controlled trials could provide more definitive evidence.

## Data Availability Statement

The raw data supporting the conclusions of this article will be made available by the authors, without undue reservation.

## Ethics Statement

The studies involving human participants were reviewed and approved by Ethics Committee Tohoku University Graduate School of Medicine. The patients/participants provided their written informed consent to participate in this study.

## Author Contributions

RN and KS conceived and designed the experiments, performed the experiments, and conducted data collection. ZW, SN, and NW sorted out the data. ZW and YK analyzed the data and wrote the manuscript. RN contributed to the devices, materials, and analysis tools. RN, ZW, and SN revised the manuscript. All authors contributed to the article and approved the submitted version.

## Funding

This work was supported by the Center of Innovation Program from Japan Science and Technology Agency, JST.

## Conflict of Interest

The authors declare that the research was conducted in the absence of any commercial or financial relationships that could be construed as a potential conflict of interest.

## Publisher's Note

All claims expressed in this article are solely those of the authors and do not necessarily represent those of their affiliated organizations, or those of the publisher, the editors and the reviewers. Any product that may be evaluated in this article, or claim that may be made by its manufacturer, is not guaranteed or endorsed by the publisher.
