# Supplementary material for: Low Back Pain Exacerbation Is Predictable Through Motif Identification in Center of Pressure Time Series Recorded During Dynamic Sitting
Source: Front Physiol. 2021 Sep 14;12:696077. doi: 10.3389/fphys.2021.696077 (PMC8476954; doi:10.3389/fphys.2021.696077)
Supplement: Supplementary file 1 [file Presentation_1.pdf]

```
# SSA-PNN
SSAPNN(dim = 5, bound = 10, max_iteration = 3, pop_size = 10,
       r_a = 1, p_c = 0.7, p_m = 0.1)

# Extreme Gradient Boosting
XGBClassifier(base_score=0.5, booster='gbtree', colsample_bylevel=1,
       colsample_bynode=1, colsample_bytree=0.5, gamma=0, gpu_id=-1,
       importance_type='gain', interaction_constraints='',
       learning_rate=0.2, max_delta_step=0, max_depth=5,
       min_child_weight=4,  monotone_constraints='()',
       n_estimators=120, n_jobs=-1, num_parallel_tree=1,
       objective='multi:softprob', random_state=8233, reg_alpha=1,
       reg_lambda=0.001, scale_pos_weight=27.400000000000006,
       subsample=0.5, tree_method='auto', use_label_encoder=True,
       validate_parameters=1, verbosity=0)

# K Neighbors Classifier
KNeighborsClassifier(algorithm='auto', leaf_size=30, metric='manhattan',
         metric_params=None, n_jobs=-1, n_neighbors=5, p=2,
         weights='uniform')

# Gradient Boosting Classifier
GradientBoostingClassifier(ccp_alpha=0.0, criterion='friedman_mse', init=None,
           learning_rate=0.05, loss='deviance', max_depth=6,
           max_features='log2', max_leaf_nodes=None,
           min_impurity_decrease=0.0005,
           min_impurity_split=None, min_samples_leaf=4,
           min_samples_split=2, min_weight_fraction_leaf=0.0,
           n_estimators=60, n_iter_no_change=None,
           presort='deprecated', random_state=8233,
           subsample=0.25, tol=0.0001, validation_fraction=0.1,
           verbose=0, warm_start=False)

# Random Forest Classifier
RandomForestClassifier(bootstrap=False, ccp_alpha=0.0, class_weight='balanced',
          criterion='entropy', max_depth=5, max_features='log2',
          max_leaf_nodes=None, max_samples=None,
          min_impurity_decrease=0.01, min_impurity_split=None,
          min_samples_leaf=4, min_samples_split=7,
          min_weight_fraction_leaf=0.0, n_estimators=190,
          n_jobs=-1, oob_score=False, random_state=8233, verbose=0,
          warm_start=False)

# Ada Boost Classifier
```

```
AdaBoostClassifier(algorithm='SAMME.R', base_estimator=None,
            n_estimators=120, learning_rate=1e-07, random_state=8233)

# Ridge Classifier
RidgeClassifier(alpha=1.55, class_weight=None, copy_X=True,
           fit_intercept=False,max_iter=None, normalize=True,
           random_state=8233, solver='auto',tol=0.001)

# SVM - Linear Kernel
SGDClassifier(alpha=0.002, average=False, class_weight=None,
          early_stopping=False, epsilon=0.1, eta0=0.2, fit_intercept=False,
          l1_ratio=0.1000000001, learning_rate='adaptive', loss='hinge',
          max_iter=1000, n_iter_no_change=5, n_jobs=-1, penalty='l2',
          power_t=0.5, random_state=8233, shuffle=True, tol=0.001,
          validation_fraction=0.1, verbose=0, warm_start=False)

# Logistic Regression
LogisticRegression(C=8.024, class_weight='balanced', dual=False,
            fit_intercept=True, intercept_scaling=1, l1_ratio=None,
            max_iter=1000, multi_class='auto', n_jobs=None, penalty='l2',
            random_state=8233, solver='lbfgs', tol=0.0001, verbose=0,
            warm_start=False)

# Linear Discriminant Analysis
LinearDiscriminantAnalysis(n_components=None, priors=None, shrinkage=0.8,
               solver='lsqr', store_covariance=False, tol=0.0001)
```
